# Kinetic Control of Ultrafast Transient Liquid Assisted Growth of Solution‐Derived YBa_2_Cu_3_O_7_‐x Superconducting Films

**DOI:** 10.1002/advs.202203834

**Published:** 2022-09-18

**Authors:** Silvia Rasi, Albert Queraltó, Juri Banchewski, Lavinia Saltarelli, Diana Garcia, Adrià Pacheco, Kapil Gupta, Aiswarya Kethamkuzhi, Laia Soler, Julia Jareño, Susagna Ricart, Jordi Farjas, Pere Roura‐Grabulosa, Cristian Mocuta, Xavier Obradors, Teresa Puig

**Affiliations:** ^1^ Institut de Ciència de Materials de Barcelona ICMAB‐CSIC Campus UAB Bellaterra Catalonia 08193 Spain; ^2^ Departament de Química Universitat Autònoma de Barcelona Bellaterra Catalonia 08193 Spain; ^3^ GRMT Department of Physics Universitat de Girona Campus Montilivi, Edif. PII Girona Catalonia E17003 Spain; ^4^ Synchrotron SOLEIL L'Orme des Merisiers Saint‐Aubin BP 48 Gif‐sur‐Yvette 91192 France

**Keywords:** chemical solution deposition, growth from transient liquid, kinetic phase diagrams, superconducting YBa_2_Cu_3_O_7‐x_

## Abstract

Transient liquid assisted growth (TLAG) is an ultrafast non‐equilibrium growth process mainly governed by kinetic parameters, which are only accessible through fast in situ characterizations. In situ synchrotron X‐ray diffraction (XRD) analysis and in situ electrical resistivity measurements are used to derive kinetic diagrams of YBa_2_Cu_3_O_7−_ *_x_* (YBCO) superconducting films prepared via TLAG and to reveal the unique peculiarities of the process. In particular, diagrams for the phase evolution and the YBCO growth rates have been built for the two TLAG routes. It is shown that TLAG transient liquids can be obtained upon the melting of two barium cuprate phases (and not just one), differentiated by their copper oxidation state. This knowledge serves as a guide to determine the processing conditions to reach high performance films at high growth rates. With proper control of these kinetic parameters, films with critical current densities of 2–2.6 MA cm^−2^ at 77 K and growth rates between 100–2000 nm s^−1^ are reached. These growth rates are 1.5–3 orders of magnitude higher than those of conventional methods.

## Graphical abstract

Transient liquid assisted growth (TLAG) is an ultrafast, non‐equilibrium growth process governed by kinetic parameters, accessible only through fast in situ characterizations. These methods are used to build kinetic diagrams of phase evolution and growth rate which are unique guides to reach the conditions for ultrafast growth (>1000 nm s^−^^1^) and high‐performance superconducting films by combining kinetic and thermodynamic parameters.

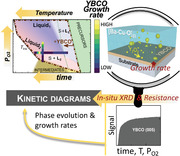

## 1 Introduction

The discovery of high temperature superconductivity (HTS) in complex oxides such as the cuprates^[^ ^1^ ^]^ has enormously boosted the interest in growing an incredible range of transition metal oxides displaying a wide span of remarkable functional properties (magnetic, semiconducting, ferroelectric, multiferroicity, catalytic, ionic conductivity, etc.). A formidable progress has been made in creating novel techniques for epitaxial thin film growth characterized by different degrees of crystalline quality, compositional control, growth rate, etc.^[^ ^2^, ^3^, ^4^ ^]^ Actually, the selection of the more adequate growth technique depends on the specific demands of each application. The discovery of HTS opened the possibility of developing many unique electrical, magnetic, and electronic applications.^[^ ^5^ ^]^ For that reason, enormous efforts are being made to develop practical superconductors having at the same time high performance and low production cost to be able to meet the industrial demands.^[^ ^6^, ^7^ ^]^

In this context, HTS coated conductors (CCs), that is, HTS REBa_2_Cu_3_O_7_ (REBCO, “RE”: rare earth) epitaxial films grown on buffered metallic substrates, appear to be a very promising approach to fabricate kilometers long superconductors. Several growth techniques of REBCO films are pursuing these goals, for instance: Pulsed Laser Deposition (PLD), Metalorganic Chemical Vapor Deposition (MOCVD), e‐beam evaporation (EV), Chemical Solution Deposition (CSD).^[^ ^8^ ^]^ One of the parameters strongly promoting cost reduction is achieving high throughput manufacturing, and this can be achieved through high growth rate methods. In this work, we investigate the recently reported ultra‐fast Transient Liquid Assisted Growth (TLAG‐CSD),^[^ ^9^, ^10^, ^11^ ^]^ based on a liquid mediated growth process that, as we demonstrate, is able to obtain epitaxial films even at 2000 nm s^−1^ growth rates (which is between 1.5 and 3 orders of magnitude larger than conventional methods). Several attempts have been made to use liquid‐based mediated growth of YBCO^[^ ^12^, ^13^, ^14^, ^15^, ^16^, ^17^ ^]^ taking advantage of the high Y diffusivity in the liquid (typically two orders of magnitude larger than in solids). However, with the non‐equilibrium TLAG process, we obtain between 1.5 and 3 orders of magnitude larger growth rates than in the methods reported so far. The novelty with respect to other liquid‐assisted techniques lies in the transient nature of the barium cuprate liquids induced in TLAG, and in the tunability of the liquid itself as well as of the Y supersaturation through kinetic parameters.

In addition, TLAG‐CSD benefits from being a cost‐effective CSD method.^[^ ^9^, ^18^, ^19^, ^20^ ^]^ The combination of ultra‐fast growth rates and cost‐effective CSD is extraordinary to achieve high throughput fabrication of CCs at reduced costs for industrial production.^[^ ^21^, ^22^, ^23^ ^]^ Moreover, among the CSD methodologies to grow REBCO films, TLAG is based on fluorine‐free precursors which, up to now, have only achieved low growth rates.^[^ ^12^, ^24^, ^25^, ^26^ ^]^ Compared to processes that use BaF_2_ as an intermediate compound, TLAG has the further advantage of simplifying furnace designs since no toxic hydrofluoric acid is generated, so a large growth area can be easily implemented.^[^ ^20^, ^27^, ^28^, ^29^ ^]^

To determine the conditions of ultra‐high growth rates and to transfer this technology to thick films and long‐length commercial metallic substrates, one needs to advance in the understanding of the working principles of TLAG‐CSD and of the transient liquid, which we tackle in this article with in situ X‐ray diffraction (XRD) and resistivity probes.

In TLAG‐CSD, the transient liquid can be reached through two main routes: the temperature route and the oxygen partial pressure route, or T‐route and P_O2_‐route, respectively.^[^ ^9^ ^]^ In T‐route, the system is heated up at constant *P*_O2_ in the region of YBCO stability.^[^ ^30^, ^31^, ^32^ ^]^ As the BaCO_3_ reacts with CuO,^[^ ^9^, ^10^ ^]^ an intermediate BaCuO_2_‐CuO transient liquid^[^ ^12^, ^33^ ^]^ is formed and YBCO crystallizes from the Y_2_O_3_ dissolution in the liquid and Y diffusion to the substrate interface. Conversely, in the P_O2_‐route, the system is heated up at lower P_O2_ where YBCO is not thermodynamically stable, and where the BaCO_3_ elimination leads to an intermediate BaCu_2_O_2_ solid phase.^[^ ^30^, ^34^, ^35^ ^]^ Subsequently, a fast *P*_O2_ increase into the region where YBCO is stable leads to the transformation of the BaCu_2_O_2_ solid phase into the transient liquid, through which again Y_2_O_3_ is dissolved and YBCO crystallizes. Notice how all these phase transformations until REBCO growth occur in a really short time (i.e., for a 400 nm film only 200 ms are needed at 2000 nm s^−1^). In both routes, the key factor is the kinetically favored formation of the transient liquid,^[^ ^9^ ^]^ which requires fast heating rates in the T‐route, as opposed to the direct reaction of the Ba—Cu—O solid phases (or vanishing amounts of liquid) with Y_2_O_3_ to form YBCO and which results in a much slower growth.^[^ ^25^, ^36^ ^]^

Each route presents its own peculiar advantages: the P_O2_‐route offers the possibility to decouple the crystallization of YBCO from the BaCO_3_ reaction with the copper oxides (Cu*_x_* O), which enables a separate optimization of each step. Conversely, the strength of the T‐route lies in the fact that the reaction between BaCO_3_ and Cu*_x_* O can be assisted/accelerated by the concomitant formation of the Ba—Cu—O transient liquid. Therefore, TLAG is a non‐equilibrium process able to grow REBCO films governed by the RE solubility and diffusivity in the Ba—Cu—O transient liquids that up to now has demonstrated ultrafast growth rates of 100 nm s^−1^.^[^ ^9^ ^]^ In this article, we show that much higher growth rates than previously published, of even 2000 nm s^−1^, are possible using TLAG. However, we need to control, first, the ability to form the transient liquid in a region where REBCO is the stable thermodynamic phase, and, second, the instantaneous Y supersaturation conditions of the liquid to induce *c*‐axis nucleation. In other words, we need a thorough understanding of the kinetic factors governing TLAG‐CSD, and of their influence in the nucleation and growth processes.

To this end, we used in situ synchrotron XRD analysis to determine several kinetic phase diagrams, which show the coexistence of the different phases at specific temperature—P_O2_ conditions, depending on the heating ramp, liquid stoichiometry and Cu oxidation state. Compared to our first report of TLAG,^[^ ^9^ ^]^ here we study both the ternary (Ba—Cu—O) and the quaternary (Y—Ba—Cu—O) kinetic phase diagrams. The existence of the liquid is directly demonstrated by investigating the Ba—Cu—O system via XRD under the same conditions as YBCO film growth. Special attention is given to the main kinetic factors determining the BaCO_3_ reaction and subsequent phase evolutions. A relevant result of this analysis is that we experimentally generate, for the first time, kinetic phase diagrams for both systems as compared to the equilibrium phase diagram used in our first report of TLAG, and which has served as reference so far. Noticeably, we demonstrate that the TLAG transient liquid can be reached upon the melting of two, and not just one, barium cuprate phases, whose formation is governed by the copper oxidation state. For the first time, we show that by modifying the value of each kinetic parameter we can change the phase evolution (formation of crystalline intermediate Ba—Cu—O phases) and change the growth rate by several orders of magnitude. Therefore, we offer quantitative tools to control phase evolution and growth rate. In addition, we used the ultra‐fast acquisition of in situ XRD at synchrotron facilities and of in situ electrical resistivity measurements to determine the growth rates in different regions of the kinetic phase diagrams, identifying conditions for ultrafast epitaxial TLAG growth even at 2000 nm s^−1^ for the first time worldwide, while obtaining high superconducting performances. These results, which represent both a 20‐fold increase in growth rate and a fourfold increase in film thickness with comparable critical current densities compared to our first report of TLAG, together with the progress reported here in understanding the fundamental aspects of TLAG that made them possible, help establish TLAG as a consistent and reproducible growth methodology.

## 2 Results and Discussion

Pyrolyzed films of Y_2_O_3_‐BaCO_3_‐CuO and BaCO_3_‐CuO were analyzed by in situ synchrotron XRD to identify the kinetics of the phase evolution and YBCO growth in the two TLAG routes (P_O2_ and T‐route). In situ XRD offers the unique opportunity to follow the ultra‐fast phase evolution characteristic of YBCO films grown via TLAG, while the synchrotron radiation enables the detection of these phase transformations at fast time scales (few tens of milliseconds of acquisition times) even with a low amount of material, such as in the case of films.

The Ba—Cu—O ternary system is discussed first for its relative simplicity (Section 2.1) while, relying on this understanding, the Y—Ba—Cu—O quaternary system is developed later (Section 2.2). The specific phase diagrams here reported refer to the Cu‐rich initial solution composition of YBa_2_Cu_4.66_O*_x_* (i.e., 3BaO‐7CuO, 3–7 stoichiometry), unless otherwise stated. The main interest in the copper‐rich composition comes from its lower supersaturation, which is able to widen the epitaxial growth window toward lower temperatures. Results on the YBa_2_Cu_3_O*_x_* (i.e., 2BaO‐3CuO, 2–3 stoichiometry) are rather similar as shown in the Supporting Information. In Section 2.3 the growth rates associated to the quaternary Y—Ba—Cu—O kinetic phase diagram are described and correlated with those obtained from in situ resistivity results.

### 2.1 Ba‐Cu‐O Kinetic Phase Diagrams: Understanding the Formation of Transient Liquids

#### 2.1.1 Ba‐Cu‐O Phase Evolution

Although some understanding already exists on the BaCuO_2_‐CuO thermodynamic phase diagram under equilibrium conditions and certain values of P_O2_,^[^ ^34^, ^35^ ^]^ here we intend to understand the kinetic reaction of the BaCO_3_‐CuO phases in non‐equilibrium conditions, and for a wide T–P_O2_ range, where YBCO TLAG films can be grown.

**Figure 1:**
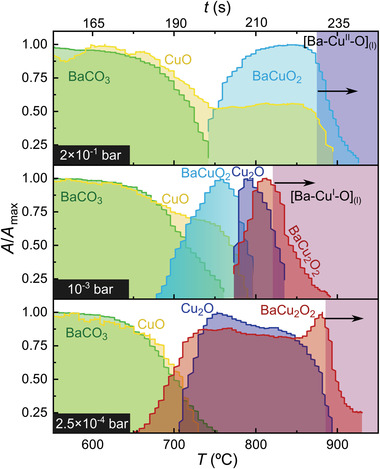
Phase evolution for Ba—Cu—O films of (3–7) composition at three different oxygen partial pressures (P_O2_ = 2 × 10^−1^ bar, 10^−3^ bar and 2.5 × 10^−4^ bar) and heating ramp of 4.5 °C s^−1^. For enhanced visibility, curves are normalized to their maximum peak area value. The black arrow indicates the non‐equilibrium melting temperature of the Ba—Cu—O phases according to the defined criterium.

The time and temperature resolved phase evolution of BaCO_3_‐CuO films are reported in **Figure** **1**, for the conditions of the T‐route at a fixed heating rate (4.5 °C s^−1^) and three different P_O2_ values: 2 × 10^−1^ bar, 1 × 10^−3^ bar and 2.5 × 10^−4^ bar. The applied heating ramp (being the maximum available with the XRD synchrotron furnace employed) is much faster than the equilibrium conditions ever investigated. However, it is well below the 20–30 °C s^−1^ employed in the laboratory studies.^[^ ^9^ ^]^

Two processes are observed for the Ba—Cu—O system in T‐route: 1) the reaction of BaCO_3_ with Cu*_x_* O; and 2) the melting of this reaction product above a certain temperature (non‐equilibrium eutectic melting). In the first process, the precursors (BaCO_3_ and CuO) react to form a barium cuprate phase. This phase corresponds to BaCuO_2_ at the highest P_O2_ (2 × 10^−1^ bar), and to BaCu_2_O_2_ at the lowest P_O2_ (2.5 × 10^−4^ bar), distinguished by the copper oxidation state (Cu^+2^ and Cu). At intermediate P_O2_ (10^−3^ bar), both the formation of BaCuO_2_ and its reduction to BaCu_2_O_2_ at higher temperature are detected.

In the second process, the disappearance of the barium cuprate and the copper oxide phases (Cu*_x_* O) reveals that melting of BaCuO_2_‐CuO^[^ ^34^ ^]^ (at 0.2 bar) and BaCu_2_O_2_^[^ ^35^ ^]^ (at 10^−3^ and 2.5 × 10^−4^ bar) has occurred in very short times (a few seconds). The melting temperature is defined as the temperature where the BaCuO_2_ or BaCu_2_O_2_ XRD integrated signal decreases by 10% of its original value (Figure 1). Although the melting of both of these phases has already been comprehensively studied in equilibrium conditions for the Ba—Cu—O and Y—Ba—Cu—O system in powders,^[^ ^30^, ^35^ ^]^ here we intend to characterize the non‐equilibrium transient liquid employed in the TLAG process.

As a consequence, the transient liquid is able to originate from both the melting of BaCuO_2_‐CuO and BaCu_2_O_2_ phases. It is known that the barium cuprate liquid originating from Cu(II) is (2BaCuO_2_‐CuO)_L_;^[^ ^35^ ^]^ while that originated from Cu(I) comes from the melting of BaCu_2_O_2_ which can occur without an additional uptake of copper.^[^ ^30^, ^35^ ^]^ However, in Figure 1, we show that some Cu_2_O can also be incorporated in the Ba—Cu(I)—O based liquid (BaCu_2_O_2_+*x*Cu_2_O)_L_. In particular in this case, a liquid with the stoichiometry (3BaCu_2_O_2_ +½Cu_2_O)_L_ is formed. However, the study of the maximum amount of Cu_2_O that can be incorporated in the BaCu_2_O_2_ liquid and its influence on the TLAG process would require further studies.

#### 2.1.2 Ba‐Cu‐O Kinetic Phase Diagram

**Figure 2:**
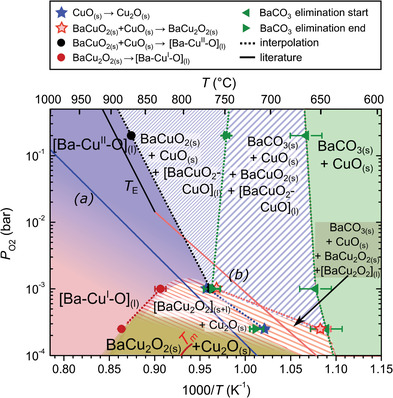
Corresponding kinetic phase diagrams based on the experiments shown in Figure 1, where the phases coexisting in each region are labelled based on the reactions indicated in the legend. Solid lines refer to equilibrium reactions from literature a) CuO_(s)_‐Cu_2_O_(s)_;^[^ ^37^ ^]^ b) BaCuO_2(s)_‐BaCu_2_O_2(s)_;^[^ ^35^ ^]^ *T*_E_: BaCuO_2(s)_+CuO_(s)_ – [BaCuO_2_+CuO]_(l)_;^[^ ^35^ ^]^ *T*_m_: BaCu_2_O_2(s)_ – [BaCu_2_O_2_]_(l)_.^[^ ^30^ ^]^ The different regions and symbols are explained in the text.

The time‐resolved phases of Figure 1 are represented in the T–P_O2_ kinetic phase diagram of **Figure** **2**. We have identified regions with the coexistence of different phases. The symbols (closed circles) at the three different P_O2_ values, connected by dotted lines, correspond to the crystalline reactions observed during the XRD experiments of this work and are shown in the legend of Figure 2; the solid lines represent the main equilibrium processes from literature experiments performed on mechanically mixed powders of the same precursors (BaCO_3_/BaO, CuO, Y_2_O_3_): eutectic melting of BaCuO_2_‐CuO (*T*_E_), CuO‐Cu_2_O reduction (a), BaCuO_2_‐BaCu_2_O_2_ reduction (b), and BaCu_2_O_2_ melting (*T*_M_).

The solid green area represents the region of the phase diagram where only solid phases coexist. It covers the initial state of the system after pyrolysis, consisting of BaCO_3_ and the yttrium and copper oxides, their recrystallization and coarsening, until their reaction starts forming a liquid.

*Region Where Liquid Ba‐Cu‐O Coexists with Solid Phases*: The striped areas correspond to regions where the Ba—Cu—O liquid can be formed and coexist with solid phases. It starts where the BaCO_3_ starts to be eliminated (10% has decomposed) and ends at the full melting (90% has melted) of the barium cuprates, as detected by in situ XRD. They represent regions where the occurrence of kinetically‐driven processes in the quaternary system leads to the coexistence of solid and liquid phases facilitating the fast TLAG YBCO growth. The idea behind these striped (solid + liquid) regions hinges upon the fact that the transient liquid can appear due to the out‐of‐equilibrium conditions imposed by the high heating ramps of the TLAG system. In fact, as we will show in the Y—Ba—Cu—O phase diagrams of Section 2.3, along this region YBCO grows at rates of 5–30 nm s^−1^ under heating ramps of 4.5 °C s^−1^, which is the highest heating rate achievable during in situ XRD experiments, but about five times below our optimal laboratory heating rates of 20–30 °C s^−1^. Additionally, the homogeneous distribution of small nanocrystalline precursors of the pyrolyzed film obtained from solution deposition contributes to speed up the reactions. This can be helped by the presence of some of the precursor phases in amorphous state (see, for example, in Figure S1, Supporting Information, the increase in the peak intensity of BaCO_3_ sometimes observed before its decrease, which indicates that some BaCO_3_, initially in the amorphous state, crystallizes). Notice that the non‐equilibrium transitions occurring in TLAG are strongly shifted from the equilibrium solid lines from literature,^[^ ^30^, ^35^ ^]^ indicating the kinetic power of the process.

*Full‐Liquid Region*: Beyond the solid + liquid regions of Figure 2, Ba—Cu—O and Cu*_x_* O solid phases have disappeared indicating that only liquid phases exist. Within this region, the liquid composition in terms of Ba—Cu—O stoichiometry will vary to accommodate for the variations in the Cu oxidation state observed in the delimiting regions. We believe this is a continuous variation from high *P*_O2_ (Ba—Cu—O liquid with a Cu(II) oxidation state) to low *P*_O2_ (Ba—Cu—O melt with Cu(I) oxidation state), thus the full liquid region is color graded in between those two extremes (Figure 2). As it is shown later, this full‐liquid region is of primary importance for the ultra‐fast growth of TLAG films.

In several binary equilibrium phase diagrams (Pb/Al, Al/In, Cu/Pb, etc.), at certain conditions of composition and temperature (and P_O2_ for non‐metallic systems such as Cr/Cr_2_O_3_) two immiscible liquids have been reported.^[^ ^38^ ^]^ However, there is so far no indication that our two ternary melts are immiscible.^[^ ^35^ ^]^ Consequently, in the absence of any evidence, we depict one single liquid region with a continuous Cu‐valence variation in TLAG kinetic phase diagrams, rather than a miscibility gap. Correspondingly, we might also expect the properties of the liquid to change with the progressive change of its oxidation state, which should probably be reflected in the TLAG YBCO properties as well.

### 2.2 Y‐Ba‐Cu‐O Kinetic Phase Diagrams: the Routes to YBCO TLAG

With this understanding on the ternary Ba—Cu—O system, and with the aim of defining the most interesting growth regions for both P_O2_‐route and T‐route, T–P_O2_ kinetic phase diagrams are defined from the in situ XRD experiments performed on BaCO_3_‐Y_2_O_3_‐CuO pyrolyzed films.

#### 2.2.1 T‐Route

**Figure 3:**
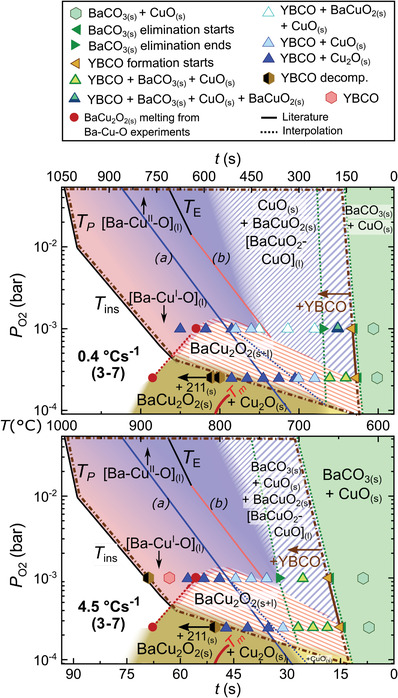
T‐route phase diagrams for the Y—Ba—Cu—O system with Ba/Cu = 3/7 ratio, measured at two different heating rates: 0.4 (upper panel) and 4.5 °C s^−1^ (lower panel). Solid lines from literature refer to a) CuO_(s)_‐Cu_2_O_(s)_;^[^ ^37^ ^]^ b) BaCuO_2(s)_‐BaCu_2_O_2(s)_;^[^ ^35^ ^]^ *T*_E_: BaCuO_2(s)_+CuO_(s)_ – [BaCuO_2_+CuO]_(l)_;^[^ ^35^ ^]^ *T*_m_: BaCu_2_O_2(s)_ – [BaCu_2_O_2_]_(l)_;^[^ ^30^ ^]^ *T*_P_: YBCO peritectic melting, YBCO – Y_2_BaCuO_5_ + L;^[^ ^31^ ^]^ *T*_ins_: YBCO instability line.^[^ ^30^ ^]^ The corresponding XRD phase evolution can be found in Figure S2, Supporting Information.

Since the main kinetic parameter for the T‐route is the heating rate, two kinetic phase diagrams are constructed, each one for a different heating rate (0.4 and 4.5 °C s^−1^ in **Figure** **3**). The results illustrate the relevant effect of this parameter.

The kinetic diagrams of Figure 3 identify the phases evolution of quaternary Y—Ba—Cu—O obtained from experiments at two representative P_O2_ values for two different heating ramps. The experimental points are plotted on the background colored diagram of the ternary kinetic phase diagram of Figure 2. The phase evolution of these four experiments is represented by solid symbols connected with dotted lines. The white region of the figure denotes the area where YBCO is not stable, either because peritectic melting occurs (*T*_P_) or because decomposition to other phases takes place upon crossing the instability line (*T*_ins_). The region surrounded by the brown dotted‐dashed line is where we have seen the crystallization of YBCO, which may coexist with all the other labelled phases. The extension of the diagram to P_O2_ higher than the 2 × 10^−3^ bar (region without solid symbols) is based on the knowledge acquired from the Ba—Cu—O system (Figure 2), although further confirmation would be required in the low temperature region. The solid lines are the equilibrium transitions also shown in Figure 2 and used as guidelines. The transition from the solid + liquid regions to the full liquid is color graded to indicate that the exact position is not identified.

If we compare the quaternary Y—Ba—Cu—O with the ternary Ba—Cu—O system at the same heating rate, (4.5 °C s^−1^, Figure 3) similar regions of phase coexistence are identified, although some relevant differences appear. In the quaternary system, the intermediate BaCuO_2_ and BaCu_2_O_2_ crystalline phases are not always observed in those striped regions of coexistence of solid and liquid barium cuprate. Thus, BaCO_3_ elimination is immediately followed by YBCO crystallization. Conversely, in the Ba—Cu—O system, both BaCuO_2_ and BaCu_2_O_2_ are detected in these regions by XRD.

**Figure 4:**
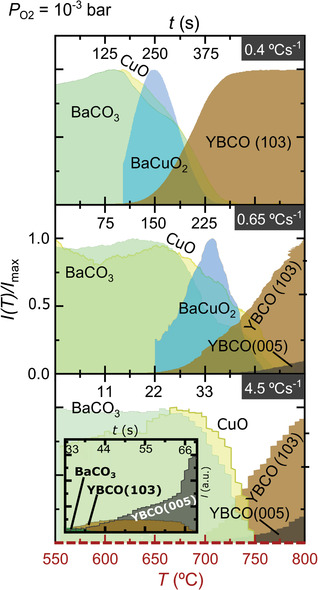
XRD phase evolution (integrated signal of the respective characteristic XRD peak) as a function of time and temperature at a fixed P_O2_ (10^−3^ bar) for the Y—Ba—Cu—O system of 2Ba‐3Cu liquid composition, comparing three values of heating rate. Each signal is normalized to its maximum value for better visibility. The dashed, red *x*‐axis at the bottom corresponds to temperature for all three cases, while the continuous black *x*‐axes correspond to the individual times. Inset: not‐normalized phase intensity showing that the YBCO (005) contribution is majoritarian.

This is further understood with the kinetic study of the quaternary phase evolution at different heating rates (Figure 3, upper versus lower panels). For instance, if we fix P_O2_ at 1 × 10^−3^ bar, BaCO_3_ elimination time (region between the two dark green triangles) is reduced from ≈100 s to just 10 s; similarly, the region of Ba—Cu—O_(s+l)_ after BaCO_3_ elimination is reduced from ≈200 s to almost zero to enter directly into the full liquid region. In other words, the system at slower heating rates (0.4 °C s^−1^) has time to crystallize some intermediate BaCuO_2_ contrarily to 4.5 °C s^−1^, where YBCO crystallizes in few seconds without the formation of the BaCuO_2_(s) intermediate. This is further illustrated in **Figure** **4**, where the time and temperature phase evolution for a 2BaO‐3CuO liquid stoichiometry is considered. Notice that, at the slower heating ramp, crystallization of the BaCu_2_O_2_ phase is observed, but it progressively disappears at higher heating ramps, until at 4.5 °C s^−1^ this phase is not detected anymore, again confirming that its crystallization is not mandatory for the YBCO growth. Therefore, we conclude that the heating rate is a very relevant kinetic parameter in the TLAG T‐route. These results also demonstrate that crystalline BaCuO_2_ or BaCu_2_O_2_ are not required intermediate phases in TLAG reaction paths, because amorphous or nanocrystalline Ba—Cu—O phases are enough to form the transient liquid.

Remarkably, YBCO nucleation is controlled through the heating rate (Figure 4). At lower ramps, homogeneous nucleation (random nuclei) is favored, whereas by increasing the heating ramp, the region where *c*‐axis nucleation is not favorable is crossed much faster, thus avoiding random nucleation and promoting heterogeneous nucleation at higher temperatures. For instance, at 4.5 °C s^−1^ (lowest panel of Figure 4), very few random nuclei are formed and most of the YBCO phase nucleates epitaxially (YBCO (005)), in this case already at the full liquid region. This can be seen in the inset of Figure 4, where the raw intensity has not been normalized and the epitaxial YBCO phase is majoritarian at the end of the heating ramp. In other words, supersaturation is indirectly modified by the heating ramp. Thus, higher heating rates are also responsible for more epitaxial nucleation. In the same inset of Figure 4, it is also shown that reorientation of the few YBCO (103) grains formed at low temperatures takes place at a higher temperature, where the region of full liquid is reached. This confirms that the liquid is able to reorient the YBCO random nuclei to epitaxial YBCO (005), as previously suggested.^[^ ^9^ ^]^

#### 2.2.2 P_O2_‐Route

**Figure 5:**
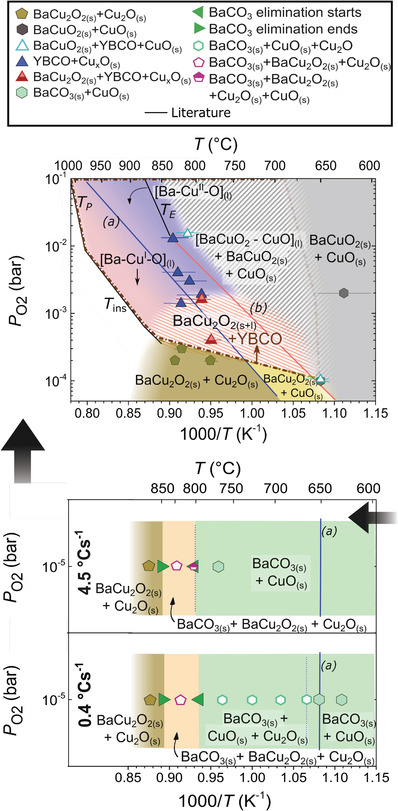
P_O2_‐route kinetic phase diagrams for the Y—Ba—Cu—O system with a 3BaO‐7CuO liquid composition. The different regions of the phase diagram are shown in different colors, the striped regions indicate coexistence of solid and liquid phases. The symbols indicate actual experiments and the color meaning is specified in the legend. The first step, at low P_O2_ (<10^−5^ bar), is shown in the lower panel for two different heating ramps. The second step, upon the P_O2_‐jump, is displayed in the upper panel. The black arrows indicate the direction of the kinetic parameter. Solid lines from literature refer to a) CuO_(s)_‐Cu_2_O_(s)_;^[^ ^37^ ^]^ b) BaCuO_2(s)_‐BaCu_2_O_2(s)_;^[^ ^35^ ^]^ *T*_E_: BaCuO_2(s)_+CuO_(s)_ – [BaCuO_2_+CuO]_(l)_;^[^ ^35^ ^]^ *T*_m_: BaCu_2_O_2(s)_ – [BaCu_2_O_2_]_(l)_;^[^ ^30^ ^]^ *T*_P_: YBCO peritectic melting, YBCO – Y_2_BaCuO_5_ + L;^[^ ^31^ ^]^ *T*_ins_: YBCO instability line.^[^ ^30^ ^]^

The kinetic phase diagram for TLAG P_O2_‐route (**Figure** **5**) is also developed from in situ phase evolution (integrated intensity of the corresponding XRD peaks). The lower panel represents the heating process at a constant P_O2_ = 10^−5^ bar, while the upper panel shows the region of YBCO growth upon the P_O2_ jump at a constant temperature. They are to be read following the arrows, which represent the direction of change in the kinetic parameter. The phase evolution of the in situ XRD experiments, at different P_O2_ values and temperatures, is represented by solid symbols, while the solid lines again represent the equilibrium transformations, as in Figures 2 and 3.

Since the P_O2_‐route consists of two steps, we will focus on a different kinetic parameter for each one. At the first stage, when heating the sample at a fixed P_O2_ = 10^−5^ bar, the main kinetic parameter is the heating rate (Figure 5, bottom panel). Notice that the CuO to Cu_2_O reduction is strongly shifted to higher temperatures as compared to its equilibrium position (blue line of Figure 5, solid and empty green hexagons), indicating the strong contribution of kinetics in all this process. In this region of low P_O2_, we notice that the reaction of BaCO_3_ with the copper oxides is delayed to higher temperatures as compared to the T‐route case. It is important to note that, depending on the furnace used, a different compromise between temperature, heating ramp, gas flow and dwell times is required to ensure the completion of this reaction before the P_O2_ jump. This BaCO_3_‐Cu*_x_* O reaction is shifted to high temperatures (850 °C, light brown pentagons) especially in the case of small, conduction‐type furnaces like the one used in this in situ XRD study, but the reaction is much faster for the tubular radiation furnaces. Despite that, the system is converted to BaCu_2_O_2_(s) and Cu_2_O(s) for both heating ramps. In this step, the microstructure and grain size of the intermediate phases will depend on this compromise: a good balance between small coarsening and BaCO_3_ elimination is needed to speed up the transient liquid formation and YBCO growth rate after the P_O2_ jump. Finally, also in this region the system deviates from the equilibrium phases expected from previously reported thermodynamic YBCO phase diagrams: neither BaCu_2_O_2_ melting is detected at this low P_O2_, nor Y_2_BaCuO_5_ (211 phase) or YBa_3_Cu_2_O*_y_* (132 phase) are observed.^[^ ^30^ ^]^ This deviation is the consequence of heating the initial precursor phases very fast.

**Figure 6:**
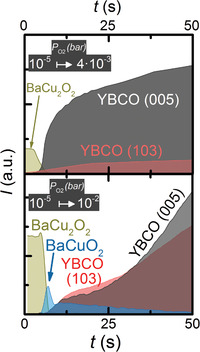
Phase evolution of two XRD experiments used to build the diagram of Figure 5 at 820 °C, showing two different situations upon the P_O2_ jump: immediate BaCu_2_O_2_ conversion to YBCO in the full liquid region (upper panel), and (lower panel) the BaCu_2_O_2_ oxidation to BaCuO_2_ prior to conversion to YBCO in the liquid + solid region.

The kinetic phase diagram upon a fast (<500 ms) P_O2_ jump is shown in the upper panel of Figure 5, and the XRD phase evolution of two specific experiments used to build this diagram are shown in **Figure** **6**. Similar regions as in T‐route are identified based on the phases observed after the P_O2_ jump, which are labelled in the legend of Figure 5: a region where no YBCO is formed upon a small P_O2_ jump, corresponding to the light brown pentagons of BaCu_2_O_2_; a region of BaCu_2_O_2_ oxidation to BaCuO_2_ with little to negligible YBCO formation, at low temperatures (gray region); and a region with the possibility of coexistence of solid and liquid barium cuprate at high temperatures (empty triangles and red triangles). This area, in P_O2_‐route, is defined by the phase evolution observed upon the pressure jump: if some crystalline BaCuO_2_ intermediate is observed (Figure 6, bottom panel), or if BaCu_2_O_2_ solid phase elimination is slowed down because there is little or no liquid produced, the system is in a solid and liquid region. Conversely, in the color‐graded regions with blue triangles (Figure 5), crystalline intermediate BaCuO_2_ is never observed upon the P_O2_ jump, and BaCu_2_O_2_ disappearance is virtually instantaneous. We attribute it to full liquid formation (or with some residual Cu*_x_* O depending on the initial solution composition). This difference is clearly revealed by the XRD phase evolution plotted in Figure 6: in the upper panel, where the jump happened in the full liquid region (blue symbols), YBCO crystallization can occur from hundreds of milliseconds to seconds, while in the lower panel, where the jump happened in the gray region where BaCuO_2_ crystallized, the growth of YBCO takes tens of seconds. More details on the criteria used to define these regions, as well as the raw XRD scans for both T‐route and P_O2_‐route, are reported in Figures S4 and S5, Supporting Information).

### 2.3 Growth Rate and Kinetic Control of TLAG‐YBCO

The interest of studying the kinetic phase diagrams lies in the opportunity to control the nucleation and growth steps of the TLAG process, and especially to identify the best conditions for the ultrafast growth rate of *c*‐axis oriented REBCO films. In this section, we will analyze the correlations of the YBCO growth rates in the different regions of the kinetic phase diagrams.

**Figure 7:**
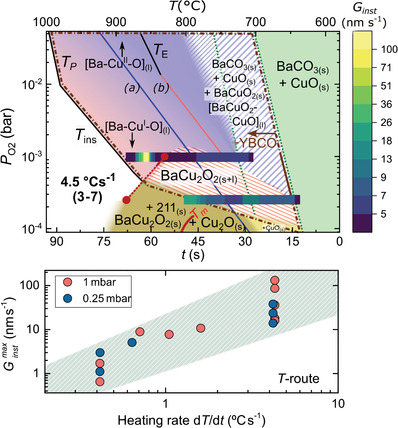
(Upper panel) growth rate phase diagram for T‐route, with the instantaneous YBCO growth rate (*G*_inst_) superimposed on the T‐route phase diagram, and (bottom panel) maximum values, $G_{\mathrm{inst}}^{\max}$ of YBCO as a function of the heating rate for two P_O2_ values. *G*_inst_ values are determined from in situ XRD.

**Figure 8:**
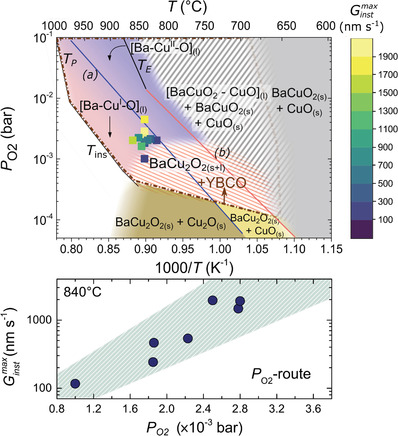
(Upper panel) growth rate phase diagram for P_O2_‐route, with the instantaneous YBCO growth rate ($G_{\mathrm{inst}}^{\max}$) superimposed on the P_O2_‐route phase diagram, and (bottom panel) $G_{\mathrm{inst}}^{\max}$ of YBCO as a function of the P_O2_, at a fixed temperature of 840 °C. *G*_inst_ values are determined from in situ electrical resistivity.

The kinetic study being our main aim, we will compile the instantaneous growth rates, *G*_inst_, calculated from the time derivative of the in situ YBCO (005) reflection integrated signal (examples in Figure S6, Supporting Information). Results of *G*_inst_, expressed in nanometers per second, are plotted on the kinetic phase diagrams of **Figure** **7**, upper panel, for the T‐route. Notice that *G*_inst_ increases until it reaches a maximum value and then it decreases when approaching full YBCO conversion. It is also noticeable that the largest values are achieved at higher P_O2_, where YBCO is still stable at higher temperatures. In the case of the P_O2_‐route (**Figure** **8**), *G*_inst_ is determined from the time derivative of the inverse of the in situ electrical resistance curve. Notice, that the inverse of the in situ resistance increases over time during the pressure jump, until it reaches saturation when the film is fully grown (see Figure S7, Supporting Information). In situ resistivity is a very useful technique to follow the growth of the superconducting YBCO phase given the large difference in resistivity between the precursor phases (insulating) and YBCO (rather metallic at growth conditions).^[^ ^39^, ^40^ ^]^ This technique was previously used by several groups to study nucleation and growth of TFA‐YBCO films.^[^ ^40^, ^41^, ^42^ ^]^ In this work, it allowed us to monitor the YBCO growth kinetics in our in‐house optimized furnaces, without the need to use in situ XRD radiation at a synchrotron facility. From this point of view, it is a fast tool to complement the information retrieved from in situ XRD analysis.

For the T‐route, *G*_inst_ values, obtained from in situ synchrotron XRD, are plotted in Figure 7 as a function of temperature, and its corresponding time scale. The maximum value, $G_{\mathrm{inst}}^{\max}$, corresponds to the maximum growth rate of the experiment which is also plotted in Figure 7, lower panel, as a function of the heating rate. For the P_O2_‐route case, *G*_inst_ values are obtained from the inverse of the electrical resistance curves obtained during growth, and the maximum values $G_{\mathrm{inst}}^{\max}$ are plotted in Figure 8, lower panel, as a function of the final P_O2_ of the experiment.

In both routes, the highest heating rates (yellow symbols in the upper panels of Figures 7 and 8) are achieved in the full liquid region of the Ba—Cu—O phase diagram, while more moderate, though still very high growth rates, are achieved in the regions were both liquid and solid barium cuprate phases coexist, confirming again the idea that enough liquid is formed in these regions to enable TLAG growth.

In T‐route (Figure 7, lower panel), the growth rate determined from the intensity of the YBCO (005) diffraction peak strongly increases with increasing heating rate. This is understood as a consequence of the fact that the system is driven further into the full liquid region. For example, by increasing the heating ramp by one order of magnitude, the growth rate can be increased by two orders of magnitude, reaching 100 nm s^−1^ at 4.5 °C s^−1^ (Figure 7). Therefore, we conclude that previous YBCO growth studies using fluorine‐free precursors but applying small heating rates, were very likely only achieving small growth rates,^[^ ^12^, ^24^, ^25^, ^26^, ^36^ ^]^ in agreement with our trend. Additionally, it suggests that, at our laboratory heating rates of 20–30 °C s^−1^, the growth rate could be much higher, possibly reaching 1000 nm s^−1^.

In P_O2_‐route, the intrinsic kinetic parameter is the fast oxygen pressure increase which brings the system to cross the YBCO instability line from the BaCu_2_O_2_ solid phase. The P_O2_ jumps associated to the points in Figure 8 (at a fixed temperature) range from 100 to just 20 ms. Consequently, in P_O2_‐route, ultrafast growth rates are associated to fast P_O2_ jumps to the region of full liquid. Here, very small changes in the final P_O2_ (by just doubling or tripling the P_O2_ value at a fixed temperature, Figure 8, bottom panel) can result in one order of magnitude differences in growth rates. The importance of acknowledging these kinetic parameters is such that much faster growth rates than previously claimed are actually demonstrated in TLAG when the kinetic state of the system is properly tuned. In fact, YBCO instantaneous growth rates as high as 2000 nm s^−1^ have been demonstrated with in situ resistivity measurements in the P_O2_‐route. We should remind here that similar YBCO growth rates have been already previously demonstrated when growing melt textured YBCO ceramics based on similar Ba—Cu—O liquids.^[^ ^43^, ^44^ ^]^

To confirm these ultra‐fast growth processes observed with an indirect method, a sample grown at ≈1000 nm s^−1^ in P_O2_‐route, was quenched at the very same moment in which the pressure jump was performed. This was achieved by cooling down the system at 100 °C s^−1^ (for the first 10 s). The TEM and XRD analysis of this sample confirmed that the YBCO layer was fully epitaxially grown (Figure S8, Supporting Information), indicating that the in situ resistance measurement is indeed representative of the YBCO growth process.

**Figure 9:**
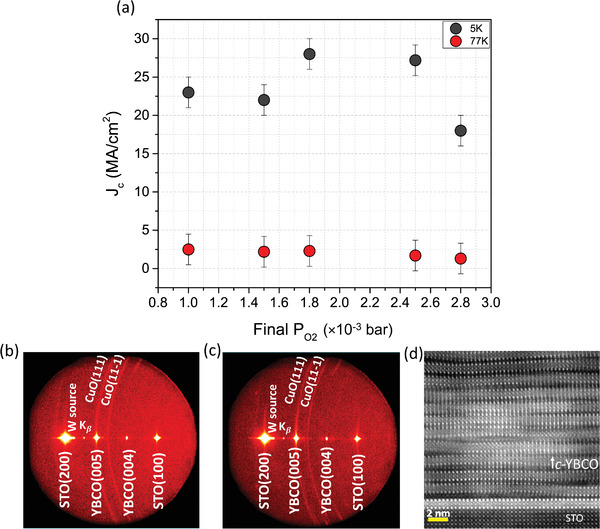
a) *J*_c_ values for a set of 400‐nm YBCO samples grown via TLAG in the range 100–2000 nm s^−1^ at different conditions of final P_O2_, showing the high performance of the process; b,c) Ex situ 2D‐XRD of two of the samples shown in (a), specifically for the conditions of P_O2_ = 10^−3^ bar and P_O2_ = 2.8 × 10^−3^ bar; d) Cross‐sectional HR‐STEM image of *c*‐YBCO/STO interface. More characterization is shown in Figure S9, Supporting Information.

Finally, results from fully grown and oxygenated films at growth rate conditions in the range 100–2000 nm s^−1^ (shown in **Figure** **9**), confirmed that epitaxial and high performance YBCO films are obtained at these high growth rates. This confirms the outstanding opportunities of TLAG and the interest for Coated Conductors manufacturing. In particular, the XRD analysis showed that epitaxial films are achieved in this P_O2_ range (see Figure 9b,c for two cases), which are also highly textured in‐plane (full width half maximum Δ*ϕ* = 0.61, not shown here) and out‐of‐plane (Δ*ω* = 0.18°, shown in Figure S9, Supporting Information). Additionally, a sharp and high superconducting transition temperature and high critical current densities were obtained (see Figure S9, Supporting Information, for a particular case with *T*_c_ = 91.7 K and Δ*T* = 5 K, and critical current densities of *J*_c_ (77 K) = 2.6 and *J*_c_ (5 K) = 23 MA cm^−2^). In this case, a charge carrier density, *n*_H_ (100 K) = 3.61 × 10^21^ cm^−3^, was measured which is representative of an optimally doped film.^[^ ^45^ ^]^ Electron microscopy results (HR‐STEM and scanning electron microscopy [SEM] images) of this representative sample, shown in Figure 9d and Figure S9a, Supporting Information, respectively, confirmed a smooth surface below the CuO surface segregated precipitates (which do not disrupt current percolation), a high quality epitaxial growth and very low porosity, all being characteristic features of liquid assisted growth.

## 3 Discussion

TLAG is an innovative growth process that takes place in conditions very far from equilibrium, contrary to many other materials processing techniques. To be able to control such a kinetically‐driven process, it is crucial to identify the nature of the liquid phase and understand the effects of the main kinetic parameters that bring the system out of equilibrium. Additionally, since TLAG is defined by the formation of a transient liquid, it is mandatory to characterize this melt across the wide T–P_O2_ region where it forms.

The transient liquid of TLAG is a Ba—Cu—O melt whose initial composition is determined by the initial solution composition (3–7 and 2–3 for this article), but whose oxidation state varies depending on the T—P_O2_ region. Thus, the liquid phase is able to change in a continuous way from [BaCuO_2_ + CuO] (l) to BaCu_2_O_2_ (l), with the Cu oxidation state continuously going from Cu(II) to Cu(I). We realized that all these melts accept large quantities of copper oxides (Cu*_x_* O), enabling to obtain a full liquid in the wide range of precursor composition employed. Therefore, we envisage a large number of factors influencing the ultrafast growth of epitaxial TLAG films. First, the amount of liquid generated at the growth conditions (T and P_O2_) is seen to have a crucial role, since all the highest growth rate values are reached in the full liquid region; thus, solid phases probably slow down the growth process. Second, high supersaturation conditions, usually ascribed to high nucleation densities, are expected to induce high growth rates.^[^ ^46^, ^47^ ^]^ In liquid assisted processes to grow YBCO (like liquid phase epitaxy and derived techniques^[^ ^14^, ^15^, ^16^ ^]^), supersaturation is determined by the amount of Y dissolved in the liquid, given by Equation (1):

(1) $$\Delta \mu =kTln\;\frac{C_{\delta }}{C_{e}}=kTln\left(\sigma +1\right)$$

where *C*_e_ is the equilibrium concentration of Y in the liquid, *C_*δ*_* is the actual concentration of Y in the liquid, *σ* is the relative supersaturation and Δ*μ* is the chemical potential change. However, being TLAG a non‐equilibrium process, Y dissolution in the melt is strongly influenced by the kinetic parameters like heating rate (in T‐route) or P_O2_‐jump speed (in P_O2_‐route). For instance, high heating ramps may shift Y dissolution to higher temperatures, thus shifting the high growth rates to higher temperatures. Third, the growth rate should also be influenced by the liquid properties like viscosity and Y diffusivity. Fourth, and perhaps with a major role, the continuous change of the liquid composition through the T–P_O2_ region (i.e., its Cu oxidation state) will accommodate changes in the liquid properties (viscosity, Y‐dissolution, and diffusivity) but also different intrinsic capabilities for nucleation (being different liquids). Thus, one cannot exclude that a Cu(II) liquid may also induce a higher growth rate than the Cu(I) liquid, as Figures 7 and 8 would suggest. Additionally, these kinetic phase diagrams are built starting from a particular solution composition and film thickness. These initial conditions will affect film composition, porosity and crystallinity after pyrolysis and during the TLAG process, so that the exact value of T and P_O2_ limiting these kinetic regions might shift when these parameters are changed. We also think that the particular combination of substrate and YBCO precursors can play a role on the exact phase evolution of intermediate phases, due to the different interface and strain energies for islands nucleation, which modify the nucleation barriers. Therefore, in the interest of defining the fastest regions for growth for a given substrate, one has to take into consideration the amount of liquid formed, the kinetics employed to reach the specific region of the T–P_O2_ diagram, the kinetic properties of the specific liquid and the initial solution composition, which are, all in all, connected in a rather complex manner since some processing parameters may influence several of these factors.

Finally, there are two additional aspects to be considered. On the one hand, the parameters chosen should be compatible with epitaxial growth. For example, in T‐route, faster ramps lead to less crystalline intermediate phases and higher epitaxial nucleation. In the case of the P_O2_‐route, given the fact that two separate steps take place, it is crucial to control both: the first stage to ensure BaCO_3_ elimination while avoiding phase coarsening, and the second stage to ensure ultra‐fast growth by tuning the P_O2_ jumps. On the other hand, the desirable conditions should be compatible with the substrates chosen. It is known that these Ba—Cu—O liquids are very reactive and corrosive^[^ ^48^ ^]^ especially at high temperatures, but also the substrate texture quality should not be affected by the TLAG growth conditions (especially for the growth of Coated Conductors on metallic substrate architectures). Despite the complicated kinetic nature of TLAG, we believe that the above considerations and the kinetic phase diagrams drawn constitute a guideline toward fast TLAG growth rates, and that in situ XRD and in situ resistivity measurements are fundamental tools to advance in TLAG understanding and transfer to Coated Conductors manufacturing. Moreover, we believe that with more targeted efforts, ultrafast growth rates above 2000 nm s^−1^ could be demonstrated, since no intrinsic limits have been found yet.

## 4 Conclusion

For both T‐route and P_O2_‐route of YBCO TLAG‐CSD, kinetic phase diagrams were constructed identifying the phase evolution that leads to ultrafast growth rates. In situ synchrotron XRD and in situ electrical resistivity measurements were used for this purpose. We have shown that the transient liquids used in TLAG have a varying composition: two barium cuprate phases are actually melting in the YBCO TLAG growth region. The direct evidence of the existence of the transient liquid has come from the generation of a kinetic phase diagram for the Ba—Cu—O system under the same processing conditions as the YBCO film growth. The properties of this liquid, as well as its amount, vary across P_O2_ and T regions together with the copper valence (Cu‐I or Cu‐II). Understanding the Ba—Cu—O kinetic phase diagram has been essential to disentangle the growth mechanisms of the Y—Ba—Cu—O system. For the practical purpose of being able to control this kinetically driven growth, we have identified some robust kinetic parameters (heating rate and P_O2_ jump). These kinetic parameters, together with the equilibrium thermodynamic parameters (T and P_O2_) and the initial solution composition, are combined to achieve the TLAG unique performance. We have proposed specific rules to understand the underlying mechanism guiding the achievement of ultrafast growth rates, and we have shown that the growth rate can be modified by more than 3 orders of magnitude depending on the value of the selected processing parameters (P_O2_, heating rate). In conclusion, we have demonstrated that by proper tuning of all these parameters, ultra‐fast growth rates of even 2000 nm s^−1^ (20 times higher than previously demonstrated with TLAG and 1.5–3 orders of magnitude higher than other growth methods) are reached while growing epitaxial and high‐performance YBCO films of 0.4–0.5 µm. Future work should show that the novel nature of this non‐equilibrium, liquid assisted, and thin film growth process can be extended to other functional materials.

## 5 Experimental Section

### Sample Preparation

The precursors’ chemical solutions were prepared starting from acetate salts of yttrium, copper and barium, according to Refs. [9, 10]. in a 1:1 mixture of propionic acid and anhydrous methanol with a total metal concentration of 1.5 m. Two types of solutions were prepared, with and without the addition of Y salt to the barium and copper mixture. The Ba—Cu ratio was either YBa_2_Cu_4.66_O_7−_ *_x_* (for the 3–7 composition) or YBa_2_Cu_3_O_7−_ *_x_* (for the 2–3 composition). In some cases, a pure propionate‐based solution with an amine additive was used, which enabled the authors to reach a higher thickness with nanoscale homogeneity and with high superconducting properties. Additional details about this solution preparation and amine composition cannot be disclosed due to confidentiality. The solution was spin coated on 5 × 5mm^2^ single‐crystal SrTiO3 (STO) substrates in a N_2_ glove box (humidity below 8%) and dried at 70 °C for a few minutes. For the specific cases of Ba—Cu solutions, MgO substrates were used to avoid liquid reactivity with Ti from STO. The samples were pyrolyzed in humid O_2_^[^ ^9^, ^10^ ^]^ with ramps of 3–5 °C min^−1^ to 500 °C and dwells of 5 min. The samples consist of 2 layers achieved through multi‐deposition, to reach several targeted YBCO thicknesses (from 350 to 500 nm), although thicknesses in the range of 1 µm have already been demonstrated by increasing the number of multi‐depositions.^[^ ^49^ ^]^ Grown films were oxygenated at 450 °C in a dry O_2_ atmosphere during 210 min.

### In Situ Analysis X‐Ray Diffraction

In situ XRD was performed at the DiffAbs beamline of Soleil Synchrotron (Paris, France) with a 6 circle diffractometer at a beam energy of 18 keV and equipped with an XPAD 2D hybrid pixel area detector,^[^ ^50^, ^51^ ^]^ which here allowed the authors to obtain fast acquisitions (100 ms/frame) and texture evaluation.^[^ ^9^, ^52^ ^]^ The detector was placed at a distance that enabled the visualization of all the phases of interest in a single 2D image (i.e., BaCO_3_ (111; 021), CuO (−111; 111), Cu_2_O (111), YBCO (005), YBCO (103), BaCu_2_O_2_ (103; 211), BaCuO_2_ (600)). The 2D images were converted into “Intensity versus scattering angle” diffractograms using a home‐made Python code.^[^ ^52^ ^]^ The sample was fixed on the heating plate of an Anton Paar furnace, model DHS1100, equipped with a graphite dome, capable of supplying heating ramps up to 4.5 °C s^−1^. The furnace was connected to a set of valves and vacuum systems allowing for the operations in a controlled atmosphere of partial oxygen pressure (P_O2_) and total pressure in each stage of the thermal treatment. The P_O2_ was controlled with mixtures of high‐purity dry N_2_ and synthetic air, pre‐calibrated with an O_2_‐sensor. For the T‐route, a gas flow of 0.6 L min^−1^ was used, while in the P_O2_‐route a two‐way vacuum system composed by a rotary pump and a turbomolecular pump was used to set the two necessary pressure states. Electrovalves were employed to execute the pressure jump in times <500 ms. The image acquisition time varied from 100 ms in Bragg–Brentano geometry (with respect to the YBCO (005) reflection), to 500 ms in grazing incidence (GIXRD) conditions. The latter was used to enhance the signal of polycrystalline phases during the heating ramp at P_O2_ = 10^−5^ bar in the P‐route process.

### In Situ Electrical Resistance Measurements

To measure the sample resistance during the thermal treatment that leads to the growth of YBCO, STO substrates were prepared for two‐probe contact resistance measurements by gluing silver wires of 0.05 mm (GoodFellow) with silver paint (CDS electronique) up to 900 °C. Then, the precursor chemical solution was deposited by spin coating and a pyrolysis thermal treatment was performed as in not‐contacted samples. Crystallization of these samples was performed at ICMAB facilities, in a quartz tube inserted in a tubular furnace, connected to the same vacuum and gas system used for synchrotron experiments. Resistance measurements were performed through a LabVIEW interface using a Keithley 2450 multimeter by applying a current of 100 µA during the whole growth process. Upon a fast P_O2_ increase in P_O2_‐route, the authors expect to observe a drop in the sample resistance (raw data in Supporting Information). A blank curve was also recorded for two‐probe contact STO samples without YBCO precursors (Supporting Information), to confirm that the resistance drop observed after the P jump is only due to the growth of YBCO.

### Characterization Techniques

Scanning electron microscopy (SEM) was carried out with a field emission QUANTA 200 FEG from FEI CompanyTM, equipped with energy dispersive X‐ray Spectroscopy (EDX). No further sample preparation is required because the final YBCO film is already electrically conductive after oxygenation.

For transmission electron microscopy (TEM) analysis, cross‐sectional specimens of YBCO thin films were prepared by conventional methods: slicing, gluing, tripod polishing and Ar+ ion milling using Gatan PIPS. Cross‐sectional high‐resolution TEM (HR‐TEM), high‐angle annular dark field scanning transmission electron microscopy (STEM‐HAADF), and energy dispersive X‐ray spectroscopy (EDX) were obtained using FEI Tecnai G2 F20 S‐TWIN HR(S)TEM microscope, operated at 200 kV. Gatan digital micrograph software (Gatan, Inc., Pleasanton, CA, USA) was used for image analysis. HR‐STEM images were obtained using a FEI Titan 60–300 microscope equipped with an X‐FEG gun, a CETCOR probe corrector, and a Gatan TRIDIEM 866 ERS energy filter operated in STEM mode at 300 kV.

X‐ray diffraction analysis of films ex situ after growth was carried out with a General Area Detector Diffraction System (GADDS) from Bruker‐AXS (model D8 Advance) operating with a CuK*α* beam source. Texture analyses were performed with a high resolution Discover D8 Bruker diffractometer. Inductive critical current densities (*J*_c_ at self‐field) were obtained with a commercial superconducting quantum interference device (SQUID) magnetometer from Quantum Design, equipped with a superconducting magnet of 7 T. In field transport critical current density, transition temperature *T*_c_ and Hall carrier density *n*_H_ were measured with a PPMS Quantum Design system equipped with a 9 T superconducting magnet. *T*_c_ and *n*_H_ were obtained using the van der Pauw method.

## Conflict of Interest

The authors declare no conflict of interest.

## Supporting information

Supporting Information

## Data Availability

The data that support the findings of this study are available from the corresponding author upon reasonable request.
