# Supplementary material for: Kinetic Control of Ultrafast Transient Liquid Assisted Growth of Solution‐Derived YBa2Cu3O7‐x Superconducting Films
Source: Adv Sci (Weinh). 2022 Sep 18;9(32):2203834. doi: 10.1002/advs.202203834 (PMC9661858; doi:10.1002/advs.202203834)
Supplement: Supplementary file 1 — Supporting Information [file ADVS-9-2203834-s001.pdf]

ADVANCED SCIENCE
Open Access

Supporting Information

for *Adv. Sci.*, DOI 10.1002/advs.202203834

Kinetic Control of Ultrafast Transient Liquid Assisted Growth of Solution-Derived $YBa_2Cu_3O_7$-x Superconducting Films

Silvia Rasi, Albert Queraltó, Juri Banchewski, Lavinia Saltarelli, Diana Garcia, Adrià Pacheco, Kapil Gupta, Aiswarya Kethamkuzhi, Laia Soler, Julia Jareño, Susagna Ricart, Jordi Farjas, Pere Roura-Grabulosa, Cristian Mocuta, Xavier Obradors and Teresa Puig**

Supporting Information

Kinetic control of ultrafast Transient Liquid Assisted Growth of solution-derived $YBa_2Cu_3O_{7-x}$ superconducting films

Silvia Rasi, Albert Queraltó, Juri Banchewski, Lavinia Saltarelli, Diana Garcia, Adrià Pacheco, Kapil Gupta, Aiswarya Kethamkuzhi, Laia Soler, Julia Jareño, Susagna Ricart, Jordi Farjas, Pere Roura-Grabulosa, Cristian Mocuta, Xavier Obradors, Teresa Puig**

S. Rasi, A. Queraltó, J. Banchewski, L. Saltarelli, D. Garcia, A. Pacheco, K. Gupta, A. Kethamkuzhi, L. Soler, J. Jareño, S. Ricart, X. Obradors, T. Puig
Institut de Ciència de Materials de Barcelona, ICMAB-CSIC, Campus UAB, 08193, Bellaterra, Catalonia, Spain. E-mail: teresa@icmab.es; silviara@buffalo.edu

J. Farjas, P. Roura-Grabulosa
GRMT, Department of Physics, Universitat de Girona, Campus Montilivi, Edif. PII, E17003, Girona, Catalonia, Spain

D. Garcia
Departament de Química, Universitat Autònoma de Barcelona, 08193, Bellaterra, Catalonia, Spain

C. Mocuta
Synchrotron SOLEIL, L'Orme des Merisiers Saint-Aubin BP 48, 91192, Gif-sur-Yvette, France

1. Phase evolution from in-situ x-ray diffraction (XRD)

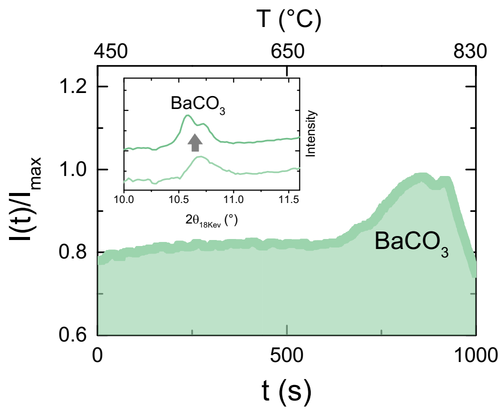

Figure S1: $BaCO_3$ phase evolution in time (determined from the evolution of the integrated intensity of the characteristic diffraction peak, see inset) during TLAG-CSD heating, showing that recrystallization of some barium carbonate can occur during the ramp up. This is an indication that amorphous material can exist before TLAG-CSD growth.

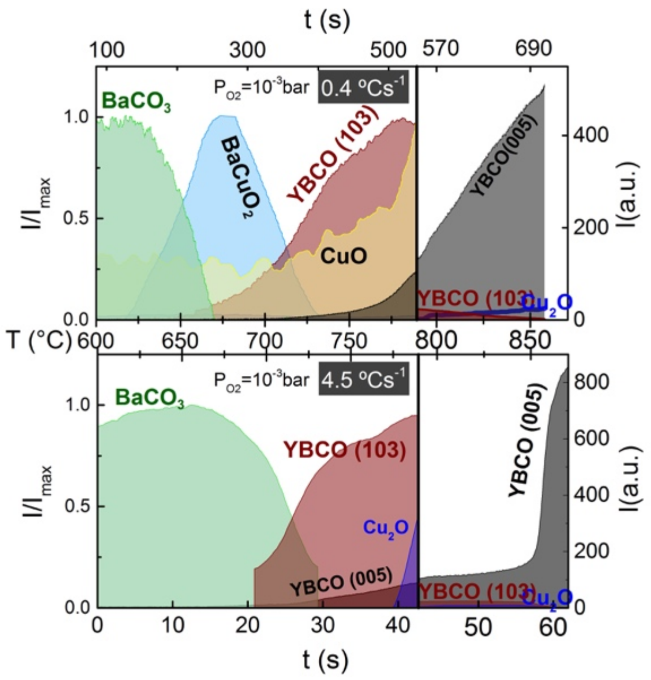

Figure S2: Phase evolution for the Y-Ba-Cu-O system with 3Ba-7Cu initial composition grown in TLAG-T-route at two different heating rates (0.4 and 4.5 °C s^{-1}). The left side axis shows the normalized diffraction intensity (I/I_{max}), while the right side one shows the raw intensity (arb. units). The corresponding T-route phase diagrams for these conditions is shown in Figure 3 of the manuscript.

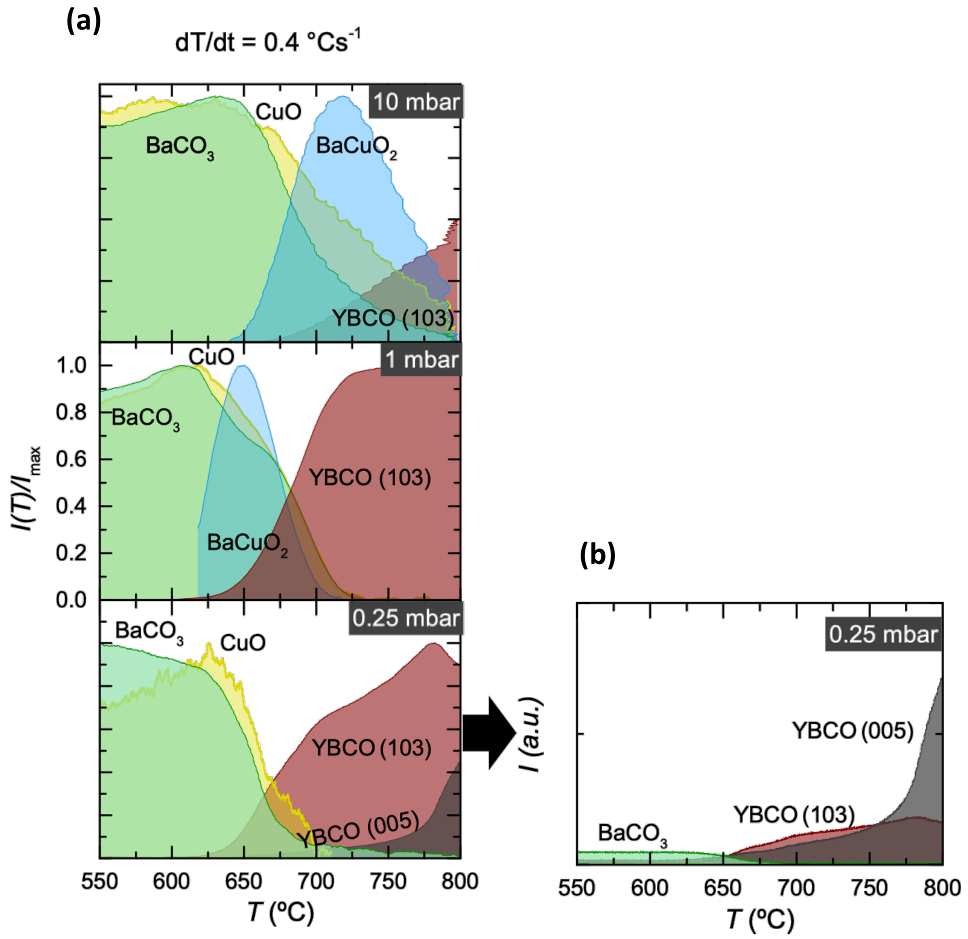

Figure S3: **(a)** Phase evolution (normalized XRD intensity) in T-route, illustrating the effect of changing P_{O2} on a Y-Ba-Cu-O film of 2Ba-3Cu initial composition. The solid intermediate Ba-Cu-O disappears as the P_{O2} decreases. In fact, this intermediate phase is not a required crystalline intermediate to form YBCO. Note that even at heating rates as low as 0.4 °C s^{-1}, epitaxial YBCO (YBCO (005)) is more intense than the random contribution (YBCO (103), as shown in the raw intensity plot in **(b)**, P_{O2} = 0.25 mbar.

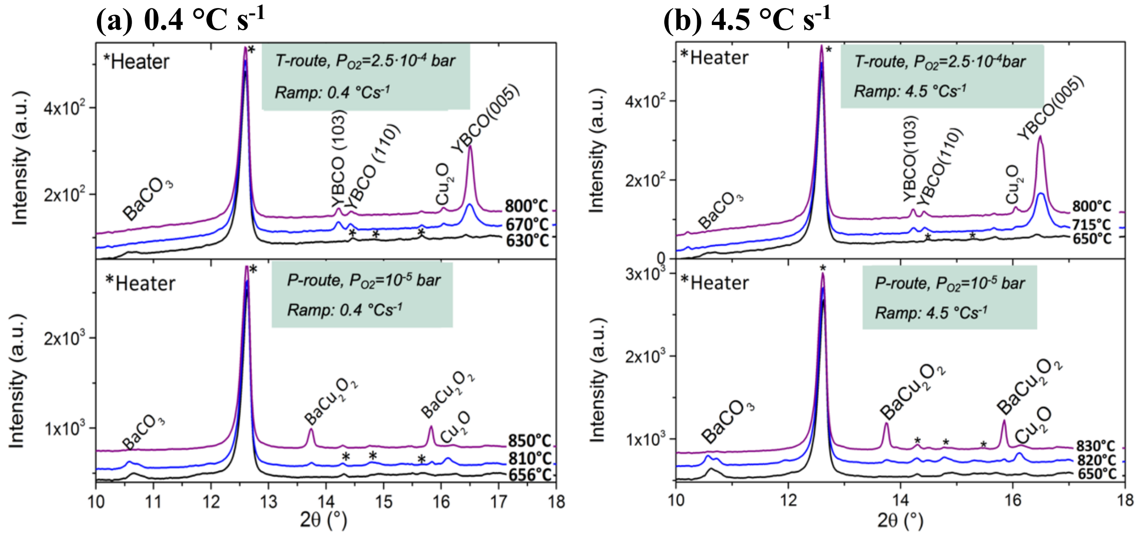

Figure S4: Example of raw data from in-situ x-ray diffraction (XRD) at two different heating rates, **(a)** 0.4 °C s^{-1} and **(b)** 4.5 °C s^{-1}. In particular: the upper panels show the integrated scans from in-situ XRD at selected temperatures during TLAG T-route in Bragg conditions with YBCO (005); the low panels show the integrated scans during TLAG P_{O2}-route at a fixed low PO_2 before the PO_2 jump, in Grazing Incidence (GI)XRD conditions. The diffraction peaks originating from the furnace (heater base plate, cover dome) used in the experimental setup are indicated by *.

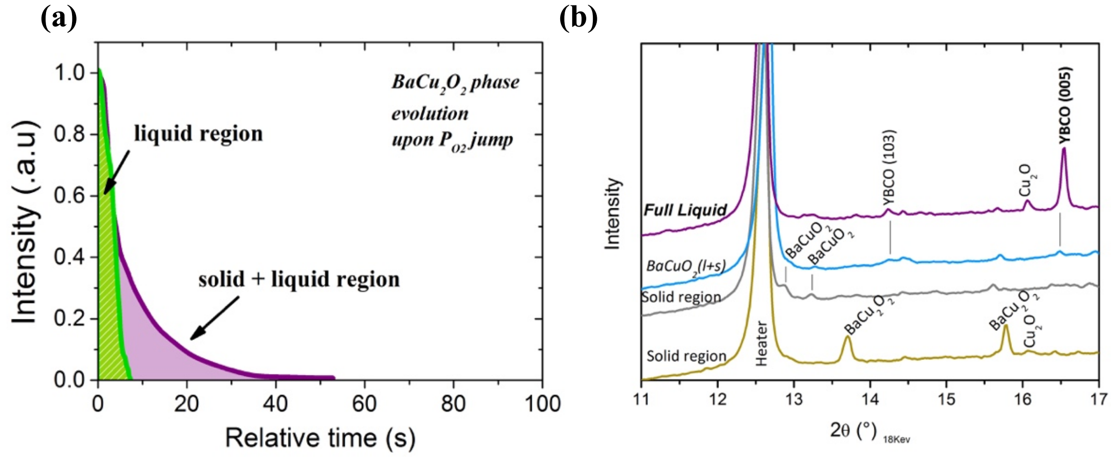

Figure S5: (a) $BaCu_2O_2$ phase evolution upon the P_{O2} jump in TLAG P_{O2}-route. Example of XRD integrated intensity evolution *vs.* time for experiments performed in two different regions of the phase diagrams, showing that the time it takes for $BaCu_2O_2$ to disappear (while YBCO is growing) is dependent on the region of the phase diagram where the P_{O2} jump is performed. (b) Example of in-situ XRD raw scans (diffractograms) of the crystalline phases detected in different regions of the phase diagrams in TLAG P_{O2}-route.

2. Growth rate diagrams from in-situ XRD and electrical resistance

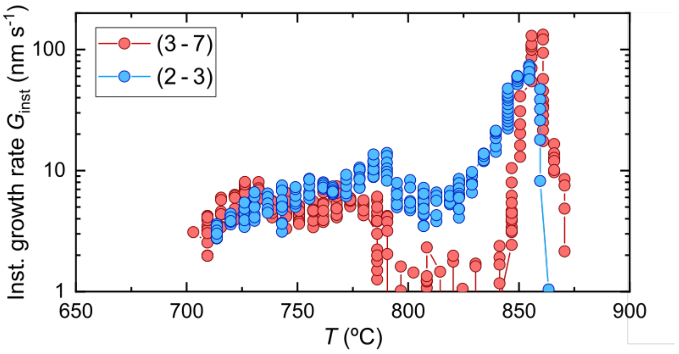

Figure S6: Derivative of the integrated signal relative to the YBCO (005) Bragg reflection for the Y-Ba-Cu-O system with 3Ba-7Cu and 2Ba-3Cu compositions. The derivative is normalized by the film thickness, and represents the growth rate as a function of temperature (or time, being time and temperature proportional in T-route). Note that G_{ins} increases until it reaches a maximum value and then it decreases again when approaching full YBCO conversion.

(a) Normalized conductance **(b)** Instantaneous growth rate

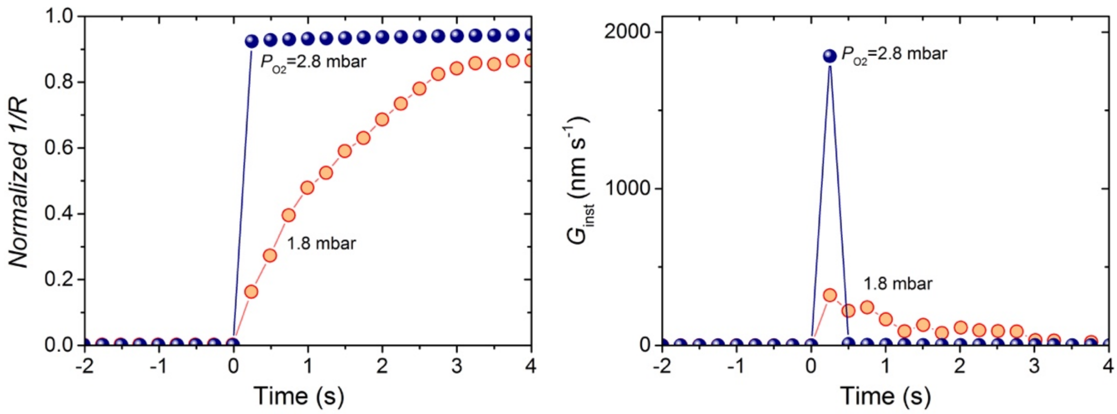

Figure S7: **(a)** Normalized conductance obtained from the inverse (1/R) of the electrical resistance curves measured in-situ during the P_{O2}-jump in TLAG- P_{O2}-route. Two different values of P_{O2} are chosen as a representative example of this class of experiments. **(b)** Corresponding instantaneous growth rate, calculated as the time derivative of the 1/R curve shown in (a) normalized to the film thickness. The highest value of (b) corresponds to G_{inst}^{max}.

(a) **(b)**

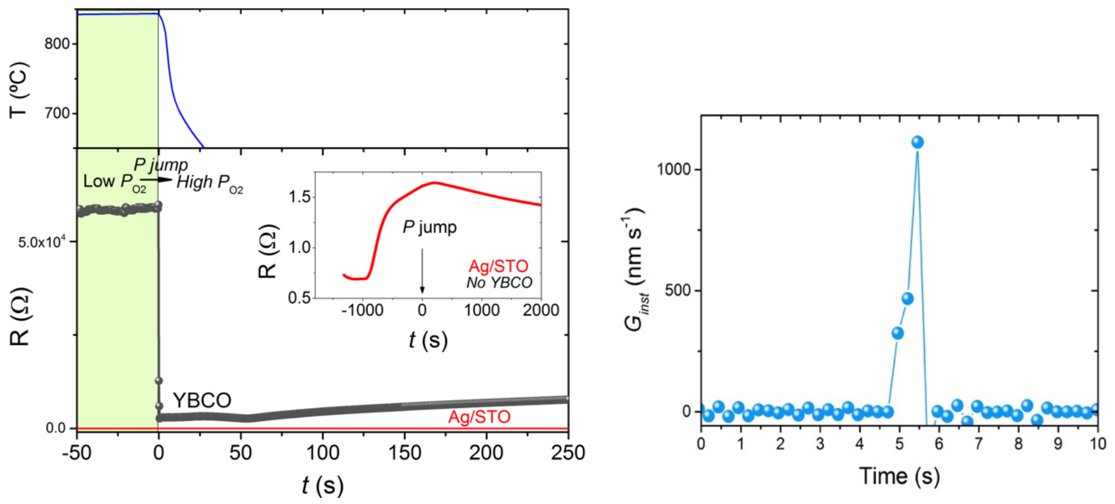

Figure S8: **(a)** In-situ resistance measurements of a YBCO sample quenched immediately at the P_{O2} change (black dots) compared to the same experiment without YBCO (red line). The effect of the silver paint resistance on the YBCO growth rate calculation is orders of magnitude less and thus it can be neglected. The quenching was performed by moving the furnace at 11 mm/s along the quartz tube, away from the sample. The temperature of the internal thermocouple placed next to the sample is reported in the top panel. **(b)** Instantaneous growth rate, calculated as the time derivative of the inverse of the resistance curve (1/R) shown in (a).

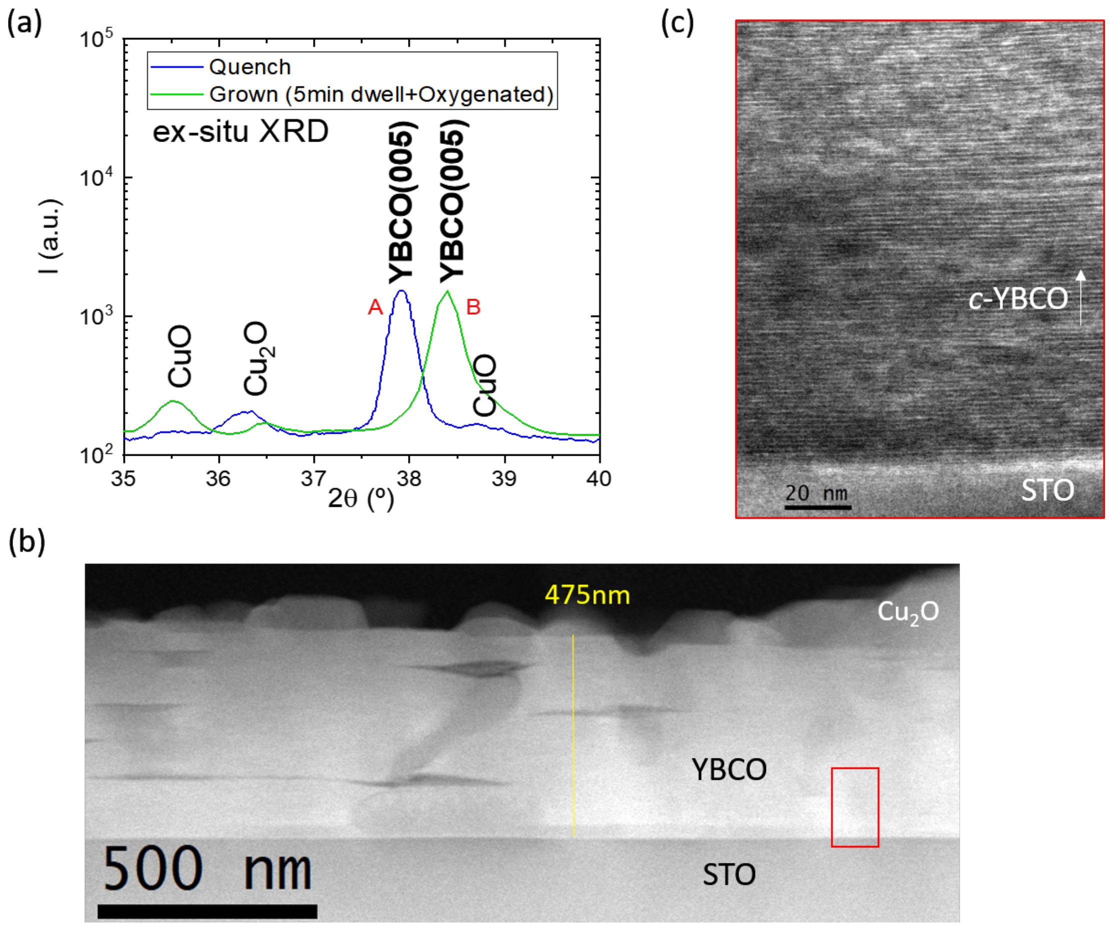

Figure S8: **(a)** XRD scan performed ex-situ on the quenched sample (A) with respect to a standard sample (B). The standard sample underwent an additional dwell time (5min) at the growth temperature (840ºC) followed by a slower cooling down and an oxygenation process. The two samples do not show significant differences in the YBCO (005) peak intensity. This confirms that the whole layer has already grown before the dwell time and that the abrupt resistance change observed upon the P_{O2} jump is representative of the growth of YBCO. The main difference among A and B comes from the oxygenation process, which shifts the YBCO (005) due to the different oxygen content, and causes oxidation of excess Cu_2O on the surface to CuO. **(b)** Cross-sectional scanning transmission electron microscopy (STEM) image of the sample, quenched immediately after the P_{O2} jump (no oxygenation), showing the YBCO film thickness ~ 500 nm and the presence of Cu_2O on the top surface. **(c)** Cross-sectional high-resolution TEM (HR-TEM) image of the YBCO/STO interface of red rectangle in (b), confirming the *c*-YBCO growth on STO.

3. Sample characterization: morphological, structural and electrical properties

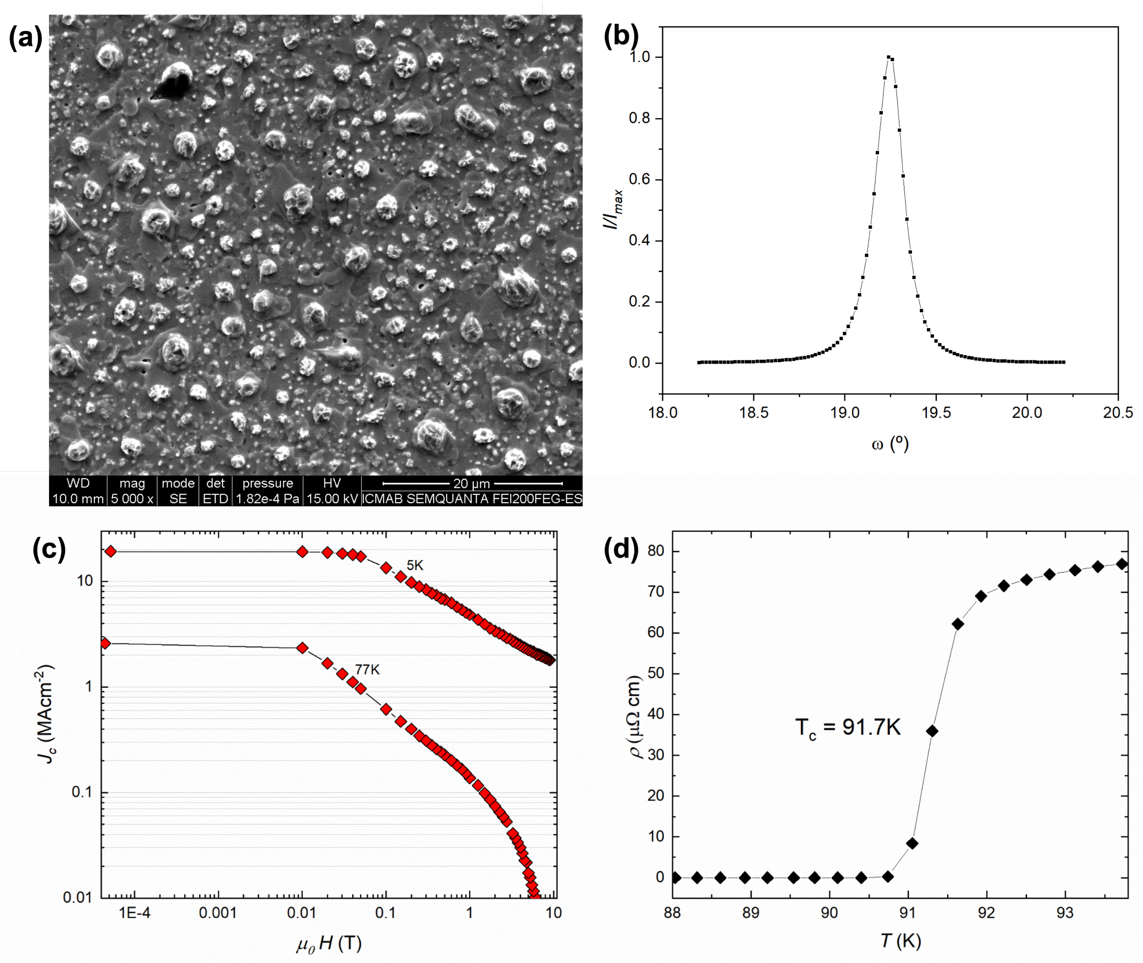

Figure S9: Characterization of a YBCO TLAG film grown with the P_{O2}-route at 840°C and 1.8×10^{-3} bar. **(a)** SEM image showing the presence of a smooth surface below the CuO particles, which come from the liquid composition with copper excess and which are pushed to the surface during the YBCO growth from the transient liquid; **(b)** shows the rocking curve for the (005) reflection of YBCO, with a FWHM value of $\Delta\omega$= 0.18º, indicative of a highly epitaxial film; **(c)** transport critical current density measured up to 9 T and **(d)** critical temperature measured in Van der Pauw geometry.
